# Contribution of S-Layer Proteins to the Mosquitocidal Activity of *Lysinibacillus sphaericus*

**DOI:** 10.1371/journal.pone.0111114

**Published:** 2014-10-29

**Authors:** Mariana Claudia Allievi, María Mercedes Palomino, Mariano Prado Acosta, Leonardo Lanati, Sandra Mónica Ruzal, Carmen Sánchez-Rivas

**Affiliations:** Departamento de Química Biológica, Facultad de Ciencias Exactas y Naturales, Universidad de Buenos Aires, IQUIBICEN-CONICET, Buenos Aires, Argentina; University of Camerino, Italy

## Abstract

*Lysinibacillus sphaericus* strains belonging the antigenic group H5a5b produce spores with larvicidal activity against larvae of *Culex* mosquitoes. C7, a new isolated strain, which presents similar biochemical characteristics and Bin toxins in their spores as the reference strain 2362, was, however, more active against larvae of *Culex* mosquitoes. The contribution of the surface layer protein (S-layer) to this behaviour was envisaged since this envelope protein has been implicated in the pathogenicity of several bacilli, and we had previously reported its association to spores. Microscopic observation by immunofluorescence detection with anti S-layer antibody in the spores confirms their attachment. S-layers and BinA and BinB toxins formed high molecular weight multimers in spores as shown by SDS-PAGE and western blot detection. Purified S-layer from both *L. sphaericus* C7 and 2362 strain cultures was by itself toxic against *Culex sp* larvae, however, that from C7 strain was also toxic against *Aedes aegypti*. Synergistic effect between purified S-layer and spore-crystal preparations was observed against *Culex* sp. and *Aedes aegypti* larvae. This effect was more evident with the C7 strain. *In silico* analyses of the S-layer sequence suggest the presence of chitin-binding and hemolytic domains. Both biochemical characteristics were detected for both S-layers strains that must justify their contribution to pathogenicity.

## Introduction

*Lysinibacillus sphaericus*, together with *Bacillus thuringiensis* var. *israelensis*, represents the best ecological insecticide against mosquitoes and an environmental friendly alternative to chemical insecticides. These Gram-positive bacteria synthesize spores together with crystal-proteins, Cry and/or Cyt toxins, both being a very stable bioinsecticide [1]. In *B. thuringiensis* var. *israelensis*, crystal Cry and Cyt proteins are involved in the recognition of the insect target, the disruption of the membrane and finally the hemolysis. This last activity has been shown to be related to the low appearance of resistant mosquitoes. Moreover, other less stable toxins are produced during the cellś vegetative growth: Vip for *Bacillus thuringiensis* [2] [3] or Mtx for *L. sphaericus* [4].

*Lysinibacillus sphaericus,* formerly *Bacillus sphaericus,* was renamed due to the presence of lysine and aspartic acid in the composition of their peptidoglycan [5]. These are a heterogeneous group of gram positive sporulating *Bacillus* some of which are entomopathogenic against mosquito larvae [4]. Hybridization studies of their DNA lead to classify them in 5 groups (I to V), but the most toxic strains belong to the homology group IIA [6] [7] and flagellar serotype H5a5b being 2362 the reference strain. These bacteria present particular metabolic traits: although they do not use hexoses or pentoses as carbon sources [8], they are able to use the amino-sugar N-acetylglucosamine, the monomer of chitin, and posses an active PTS transporter (Phosphoenolpyruvate phosphotransferase system) essentially implicated in its utilization [9] [10]. However, in contrast to *B. thuringiensis* strains [11], no chitinase activity has been detected in these bacteria.

*L. sphaericus* spores present an important exosporium allowing spore and crystals to remain firmly associated [12]. These crystalline inclusions are composed by two proteins named BinA and BinB, which can form dimer and/or associate in mixed proportions [13]. During the vegetative growth phase *L. sphaericus* strains produce several toxic proteins named Mtx1, 2, 3 [14] [15]. Besides being very efficient in synergic experiments with BinA-BinB, Mtx proteins are not synthesized during the sporulation phase and are degraded by proteases synthesized during this period. In fact, recombinants containing the cloned *mtx1* gene under a *bin* promoter allow Mtx1 synthesis during sporulation, but again the protein was rapidly degraded while sporulation proceeds [16].

While *B. thuringiensis* var. *israelensis* spore-crystal preparations are highly active against *Aedes*, *Culex*, *Anopheles* and *Simulium* [1] [4] those from *L. sphaericus* are essentially active against *Culex* and *Anopheles* species. This complementarity in behavior and targets has been exploited by using mixed preparations and recombinants containing the cloned toxic genes from *L. sphaericus* [17] [18] [19] into *B. thuringiensis*. However, *B. thuringiensis* is reported to be highly sensitive to the presence of chemical and metal contaminants, while *L. sphaericus* shows a better persistence in contaminated ponds [4]. Also several reports have shown the ability of this bacterium to survive [20] [21] and bioabsorb metal at concentrations otherwise toxic [22] and this property is linked to the presence of their S-layer envelope.

Moreover, it is worthwhile mentioning that the S-layer from several bacteria have also been implicated in their pathogenicity; this is especially so for *Bacillus* species as *B. cereus*, *B. anthracis* [23] [24], *Paenibacillus alvei* [25], different strain-variants from *B. thuringiensis* [26] [27] [28] and new isolated from *L. sphaericus* [29]. Altogether these properties have increased the interest in *L. sphaericus* strains and the drive to find new isolates of this species.

The reference strain 2362 is endowed with a high molecular weight S-layer (120 kDa) [30] that was also present in spore preparations [22]. Sequence based analysis performed for this protein allowed to predict the presence of hemolytic and chitin binding domains that might contribute to their entomopathogenicity. Such properties are associated with the pathogenicity of the Cry proteins from *B. thuringiensis* [31]. This led us to investigate the insecticidal properties of the S-layer from two *L. sphaericus* strains: the reference strain 2362 and C7 (a new isolate for which a higher insecticidal activity of the mixture of spore-crystals preparations has been observed). The mosquitocidal activity against *Culex* and *Aedes* larvae of spore-crystals and purified S-layers was assayed. Hemolytic activity and chitin binding properties of the S-layer were analyzed to characterize their influence on the mosquitocidal activity of *Lysinibacillus sphaericus*.

## Materials and Methods

### Bacterial strains, antibodies and media

*L. sphaericus* 2362 was obtained from Institut Pasteur. C7 strain was isolated from a crocodile lagoon in Cuba. Flagellar antisera (provided by A. Delécluse, Institut Pasteur) and phages typing (provided by A. Yousten) characterizations allowed us to classify them in the same group as 2362 (H5a, H5b). In addition, the antibody against the S-layer from 2362 (provided by L. Lewis) [30] recognizes the S-layer from the C7 strain. The anti-BinA or BinB were kindly provided by JF. Charles (Institut Pasteur). Strains were grown in LB (Luria Bertrani) or NYSM (Nutrient Yeast Extract Salt Medium) medium [32] as indicated in the text in aerated conditions at 32°C. For spores preparations growth was on solid NYSM medium at 32°C and spore-crystals were collected after 3 day incubation.

### Phage typing assay

The phage-typing of the isolated strain was performed as described previously and the phages used were provided by A. A. Yousten [33]. Bacteria were grown on NYSM agar at 30°C. Phages 1A and 2 were propagated on strain *Bacillus sphaericus* SSII-1; phages 12 and SST were propagated on strain Kellen K; and phages 4 and 5 were propagated on strain 1593. For typing, bacteria were seeded into NYSM soft agar overlays and 20 µl of diluted phage suspension was spotted onto the surface. Plates were incubated at 30°C for 18 h. The production of individual plaques at the same dilution or at a 10-fold more concentrated dilution than those producing individual plaques on the normal phage propagating strain was noted as a positive result. Patterns of test results were compared with those for standard strains 1593 and 2362 (phage group 3), SSII-1 (phage group 2), and Kellen K (phage group 1).

### Spores-crystal preparations

Strains were grown on solid NYSM medium and incubated 2–3 days at 32°C. Plates were then washed with 10 ml of 1 M NaCl, washed four times with milliQ H_2_O and suspended in 10 mM sodium acetate pH 4.5 that prevents germination [34]. Dry-pellets containing a mixture of spores-crystals were stored at −20°C.

### Cleaning off S-layer protein from spores

Spores-crystals from 3-day cultures were scrapped and washed with 10 ml of 1 M NaCl, then in deionized water, in 0.5 mM EDTA which eliminates the S-layer, and finally four times in deionized water [22]. These spores were used to evaluate synergy with purified S-layer proteins.

### S-layer purification from cultures

*Lysinibacillus sphaericus* 2362 and C7 strains were grown in LB medium. Cells from 100 ml exponential cultures (OD_600_ = 1) were harvested and washed once with PBS. The S-layer proteins were extracted from the cells by cationic substitution by resuspending in 10 ml of 6 M LiCl, vortexing and incubating for 30 min at room temperature. After centrifugation at 15,000×g for 15 min, the supernatant was collected and dialyzed against 10 mM CaCl_2_ overnight at 4°C. After centrifugation (10,000×g for 20 min), the pellets containing the S-layers were resuspended in deionized water and stored at −20°C [22].

### Protein analysis

Spores proteins were alkali-solubilized (30 min at 37°C in 0.05 N NaOH) [17]. After alkaline extraction the preparations were centrifuged at 12,000×g 5 min to produce Pellet (P) and supernatant (S). The spores and S-layers preparations were heated at 90°C 5 min in loading buffer (10% glycerol, 4% SDS, 4 M urea, 2% β-mercaptoethanol, and 0.05% bromophenol blue) and subjected to electrophoresis in 12.5% SDS-PAGE. Gels were stained with Coomassie Brilliant Blue. The same amount of material was loaded in each well (10^7^ CFU of spores and 0.5 µg of S-layer protein).

For western blot analysis, gels were electrotransferred with a Semi-dry Blotter (Amersham Biosciences) to PVDF membranes (Macherey-Nagel, Germany) soaked with polyclonal anti-rabbit antibodies against S-layer or BinA or BinB (diluted 1∶2000 for S-layer and 1∶50000 for BinA and BinB), and visualized with biotin-conjugated anti-rabbit followed by streptavidin-HRP conjugate (Pierce). Chemiluminescence was detected with luminol substrate (ECL from Sigma). Images were obtained with a Fuji LAS1000 digitalizer.

### Detection of S-layer protein in spores by immunofluorescence

An immunofluorescence assay was performed in order to detect the presence of S-layer protein probably associated to the exosporium as described in *B. anthracis* [35] [36]. Spores from 3-day cultures were scrapped, washed with 10 ml of 1 M NaCl and resuspended in 10 mM sodium acetate pH 4.5 that prevents germination [34]. After centrifugation and double washing with PBS, the pellets were resuspended in PBS. A 20 µl (10^8^ CFU) spore sample was then mixed with 0.5 µl antibody against S-layer and incubated 1 h at 25°C at a low shaking speed. The sample was then centrifuged, washed twice with PBS, resuspended in 100 µl PBS and subjected to the second antibody. 1.5 µl of antibody [Alexa fluor 647 goat anti-rabbit IgG (H+L) (Invitrogen)] was added and the sample was incubated for 30 min at 25°C. Spores were then pelleted, washed twice with PBS, and resuspended in 50 µl PBS. A drop was applied to a glass slide and overlaid with a coverslip. Immunofluorescence was observed with fluorescence microscopy on an Epifluorescense LED Axio Scope A1 model Microphot microscope (Carl Zeiss), N-Achroplan 100x/1.25 Oil PH3, filters mCherry FS64HE. The images were taken with a EOS T3 1100D digital camera (Canon) (10,1 Mega Pixel CMOS, 3.888×2.592 pixel eff, lens 18–55 mm).

### Larvicidal assays

Experiments were carried with autochthonous seasonal species of *Culex sp* and *Aedes aegypti* larvae supplied by “Grupo de estudios de Mosquito” of the University of Buenos Aires [37]. Eggs were spread in dechlorinated water, fed with yeast extract, maintained at 28°C with daily illumination. After 4 days the majority of larvae reached 2–3^rd^ instar development and were ready to use.

Petri dishes (10 cm diameter) containing 20 ml dechlorinated water and 20 larvae (2^nd^ and 3^rd^ instar) were inoculated with different concentrations of spores, S-layer protein or both as indicated in Tables 1 and 2 and were incubated at room temperature [38]. Larval mortality was evaluated 24 and 48 hours after exposure and compared to controls. The concentrations giving 50% mortality (LC50) was expressed as number of spores (CFU/ml) or S-layer proteins (µg/ml) after subtracting the mortality of controls. Assays were performed in duplicate and repeated three times. A representative experiment is shown.

**Table 1:** Larvicidal activity of S-layers and spores from 2362 and C7 strains against *Culex sp* and *Aedes aegypti* larvae.

	*Culex sp.*	*Aedes aegypti*
**S-layers from**	**LC50 (µg/ml)**	**LC50 (µg/ml)**
2362	2.2 (0.2)	>80
C7	2.0 (0.2)	8.0 (0.5)
**Spores from**	**LC50 (cfu/ml)**	**LC50 (cfu/ml)**
2362	1.00×10^3^ (0.02×10^3^)	1.20×10^6^ (0.06×10^6^)
C7	0.50×10^3^ (0.02×10^3^)	5.00×10^4^ (0.25×10^4^)

S-layer and spores preparations were added with 20 *Culex sp.* or *Aedes aegypti* larvae as indicated in Material and Methods. After 48 h lethality was evaluated. Assays were performed with duplicate and repeated three times and a representative experiment is shown. In parenthesis, standard deviation.

**Table 2:** Synergy between spores and S-layers against *Culex* sp. Larvae.

	2362	C7
Mortality with Spores	32%	20%
Mortality with S-layer	2%	5%
Mortality with both	62%	85%

S-layer and spores preparations were added separately or mixed at sub-lethal concentrations with 20 *Culex sp.* larvae as indicated in Material and Methods. After 24 h lethality was evaluated. Only the % of died larvae are reported. The concentration of spores or S-layer protein individually or together per experiment were 500 CFU/ml and 0.25 µg for 2362 strain and 50 CFU/ml and 0.6 µg for C7 strain.

### Binding of S-layers to chitin compounds

To determine if the S-layer proteins recognize the chitin present in the cuticulum of insects, the binding specificity of these proteins for insoluble polysaccharides was used as follows: 50 µg of purified proteins were mixed with 10 µg of polysaccharide in a total volume of 500 µl PBS and incubated for 16 h at 32°C. The samples were centrifuged at 3,000×g for 3 min to separate free protein from substrate bound protein. Protein concentrations in the supernatant were determined using Bradford (BioRad). The percentage of adsorbed protein was calculated by substracting the free protein amount from the total protein in the sample. Polysaccharides were also incubated with bovine serum albumin, BSA, as negative control.

The polysaccharides used for this experiment were powder chitin, crab chitosan and fish chitosan resuspended in H_2_O deionised and sonicated (to solubilise particles). These assays were performed at least six or more times as independent experiments in duplicate each time.

### Hemolytic assays

Hemolysis is a way to evaluate membrane perturbation, a feature present in entomopathogenic toxins [31]. % Hemolysis was determined as previously described [39]. Briefly, sheep red blood cells were separated by centrifugation (1,000×g for 5 min) and resuspended to 1% with PBS. The final volume of the reaction mixture was 1 ml containing 0.5 ml of washed blood cells and various concentrations of S-layer protein (0.5–150 µg) in the same buffer and incubated at 37°C for 30 min. After centrifugation at 1,200×g for 5 min hemolysis was quantified measuring the absorbance of the supernatant at 405 nm. Positive control was 100% hemolysis after incubation of the same volume of sheep red blood cells with dechlorinated H_2_O. Negative controls were red blood cells incubated with buffer. As a specificity control, antibodies (1∶200 dilution) against S-layer were incubated for one hour along with the S-layer protein. Subsequently, the mixture was tested for hemolysis. These assays were performed five or more times as independent experiments in duplicate each time.

### Statistical Analysis

Statistical significance was evaluated by the Mann Whitney-U test for nonparametric data by Infostat software [40]. P<0.05 was considered to be statistically significant.

## Results

### Spore-crystals preparations contain S-layers

**Figure 1:**
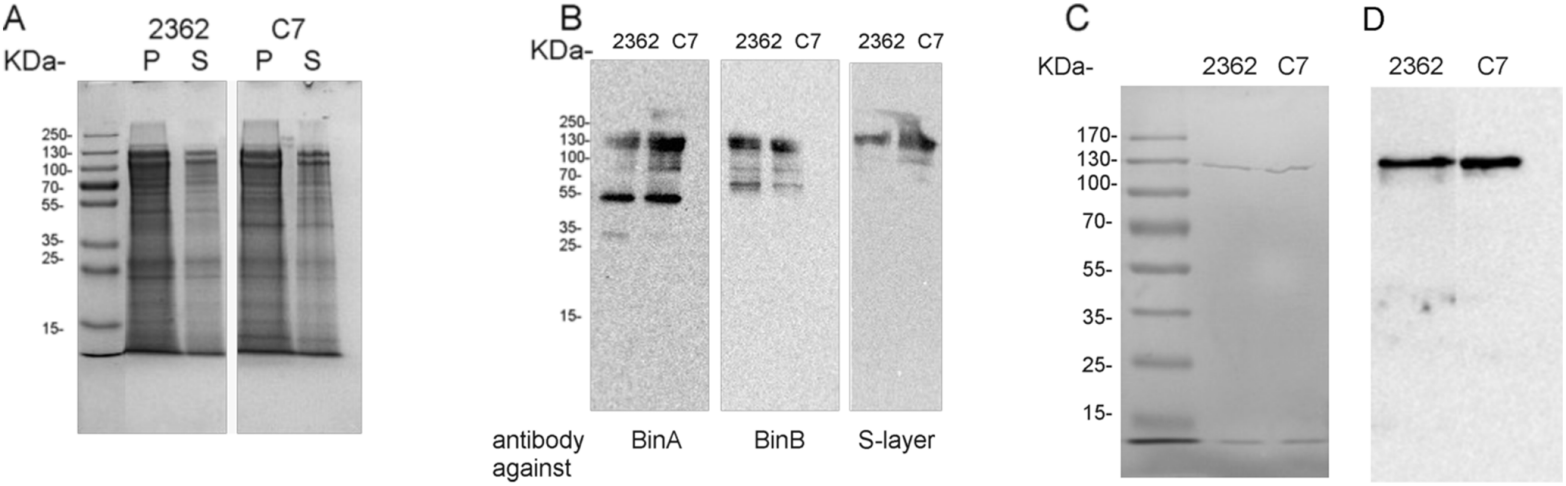
Detection of S-layers in spore-preparations and vegetative cultures. Pellet (P) and supernatant (S) fractions of spore-preparations from 2362 and C7 strains, obtained from the alkaline treatment described in Materials and Methods, were subjected to SDS-PAGE 12.5% (A) and Western Blot analysis for detection with specific antibodies against BinA or BinB or S-layer proteins (B). Purified S-layers from 2362 and C7 strains were obtained from vegetative cultures as described in Materials and Methods. 6 µg of each preparation were subjected to SDS-PAGE 12.5% electrophoresis (C) and analyses by Western Blot with specific antibody against the 2362 strain’s S-layer (D).

In a search for *Lysinibacillus sphaericus* strains with enhanced insecticidal activity, one particular isolate C7 was obtained. The C7 strain belongs to the same phage group 3 as the 2362 control. Its spore-crystals preparations were at least 100-fold more active than 2362 against *Culex pipiens* 2^nd^ and 3^rd^ instars larvae (LC_50_ as µg total protein/assay were 1500±340 and 15±2 for 2362 and C7 respectively). Moreover, the strain had the same metabolic features as the reference strain 2362: growth with N-acetylglucosamine but not with glucose as carbon source, antigenic and phage-typing characteristics, presence of Mtx and Bin in spores. Since no clear difference in the yield of Bin toxins present in spores was observed, we undertook the analysis of the S-layer content of spores. We had previously observed that S-layers remain associated to spores [22], but these proteins could be a remnant of the vegetative envelope, or a constituent of the spore itself. For this purpose two approaches were used: Western Blot and immunofluorescence. Spore-crystal preparations were analyzed and detected using Western Blot. In this condition several high molecular weight bands (>120 kDa) are usually observed. This is generally attributed to associations between the toxic components BinA and BinB [13]. Western blot analyses with antibodies against Bin toxins and S-layer protein reveal the latteŕs presence in the high molecular weight band (HMW up to 130 kDa), confirming that the S-layer and Bin components remain closely associated (Fig. 1A and B). Furthermore, the more toxic strain, C7, produced spores containing higher amounts of S-layer protein, which was also recognized by the specific antibody from 2362.

**Figure 2:**
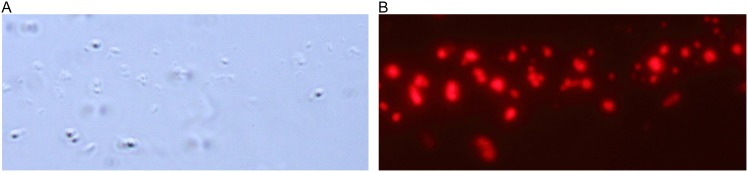
Detection of S-layers in spores by immunofluorescence. Spores preparations from C7 (A and B) were cleaned as described in Materials and Methods. A: Microscopic white light observation. B: Fluorescent (652 nm excitation and 668 nm emission) observation of the S-layer in the same preparation.

An immunofluorescence assay was used to directly detect the presence of S-layer protein in spores in association with the exosporium as has been described in *B. antracis* [35] [36] S-layer protein outside the spores, probably in association with the exosporium, was detected through direct observation of S-layer protein in non permeabilized spores (Fig. 2).

### Larvicidal activity of S-Layer proteins from vegetative cultures and spores

S-layer extracted by LiCl and analyzed by SDS-PAGE and Western Blot showed a band running as 120–130 kDa (Fig. 1C and D). These purified S-layers proteins from C7 and 2362 strains were used to feed *Culex sp*. larvae as described in Materials and Methods. The dose response mortalities were obtained. The S-layer from C7 was as efficient as that of 2362 against *Culex* larvae (LC_50_ 2.2 and 2.0 µg/ml, respectively) (Table 1). Surprisingly, when assayed against *Aedes aegypti*, only that of C7 showed a substantial activity (10-fold more toxic C7 than 2362) (Table 1). This led us to investigate possible interactions of the S-layers and spores in the larvicidal activity.

### Synergy of spores and S-layers in the larvicidal activity

For this purpose individual batches of spores-preparations from 2362 and C7 strains were analyzed in order to determine their activity in the same batch of *Culex sp*. and *Aedes aegypti* larvae. This caution is necessary since batches of mosquitoes vary between seasons and origin.

In this condition we determined the LC_50_ for each preparation (spores and S-layers) and strain (2362 and C7). In this assay (Table 1), spores from C7 were 2 and 25 times more toxic against *Culex* and *Aedes* respectively than those from strain 2362.

In order to check for synergistic effects S-layer was removed from spores with EDTA [22]. Using concentrations of spores and S-layers giving mortality below 50%, a synergistic evaluation between them was assayed (Table 2). As shown, the mixtures (spores and S-layers), at sub-lethal concentrations, had 2 to 3 times higher activity than each component on its own, indicating that a synergistic effect between spores and S-layers took place.

These findings demonstrate the importance of the S-layer protein present in spores preparations in the larvicidal activity of these bacteria.

### Sequence based analysis for putative functionalities that could support larvicidal activity in the S-layer protein

In order to characterize if a biochemical support for *Lysinibacillus sphaericus* pathogenicity is provided by it S-layer, a search of possible domains present in this protein was performed. Using different programs that are described in the Material S1 (SMART, EMBOSS Matcher Pairwise Sequence Alignment, and Clustal-O) the sequences of SlpC proteins were analyzed. AAA50256.1, surface layer protein of *Lysinibacillus sphaericus* 2362, was used. When we analyzed the SMART database to predict functionality and physical distribution, we found a similar arrangement of surface layer homology domains (SLH) and Internal Repeats as that observed for the chitinase ChiW (BAM67143.1) of *Paenibacillus sp*. FPU-7 (Fig. S1A) [41]. Homologue architecture was observed between the two proteins: three SLH domains and two Internal Repeats. A local similarity to GH 18 chitinase domains was found for the internal repeats (Fig. S1B and S1C) using EMBOSS Matcher program, which identifies local similarities in two input sequences using a rigorous algorithm and allows for the modification of default substitution scoring matrices (BLOSUM) for sequence alignment between distantly related proteins. Using Clustal-O, a global alignment tool for multiple proteins sequences, homology with ACA38715.1 hemolysin-type calcium-binding domain-containing protein (874 aa, from the complete sequence of *Lysinibacillus sphaericus* C3–41 accession number NC_010382), was obtained with a high score alignment (Fig. S1D). The predicted functionalities were then investigated using *in vitro* assays for chitin binding and hemolytic activity in these S-layers preparations.

### Chitin binding assays of S-layers

**Figure 3:**
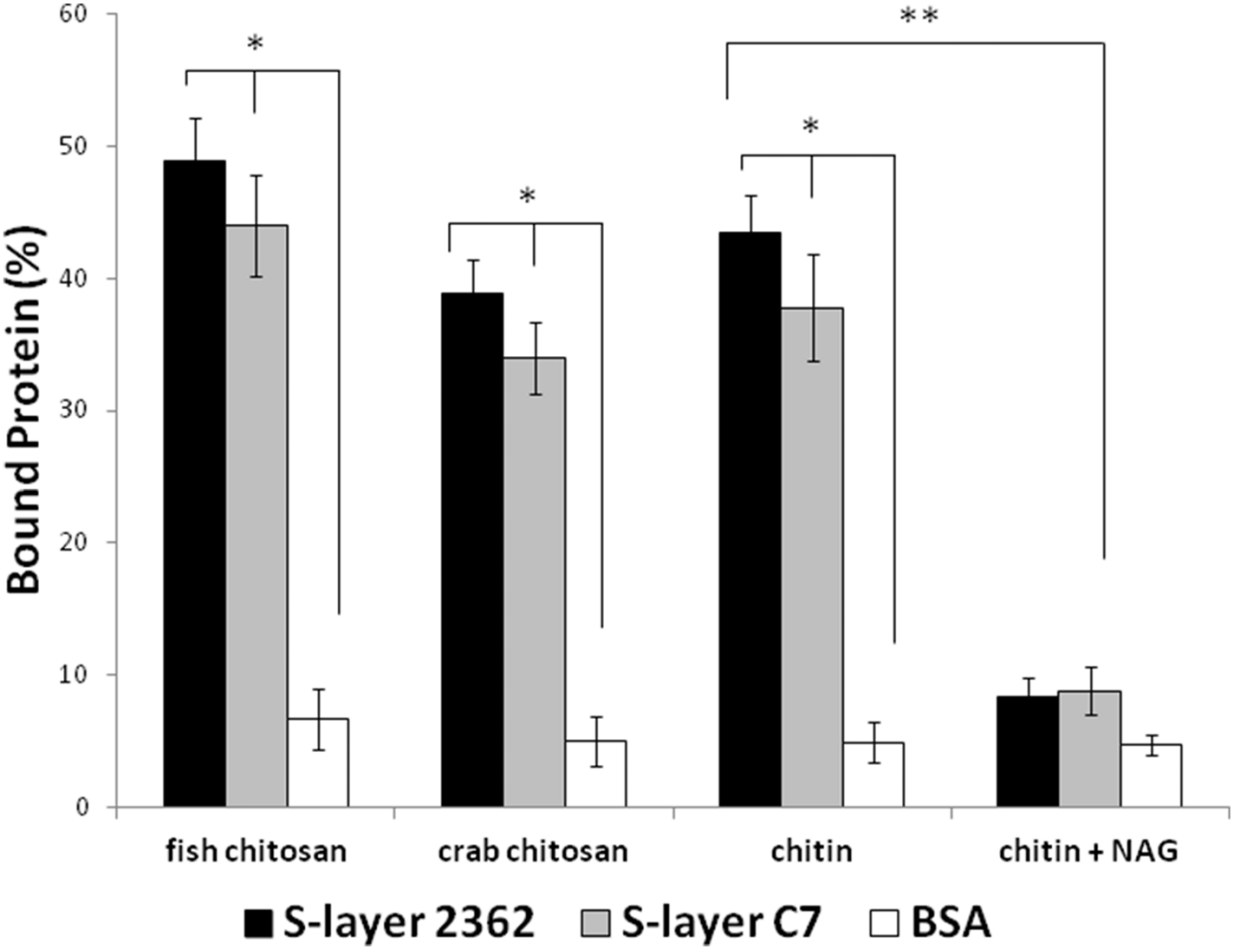
Binding assays of S-layer to chitin derivatives. Insoluble preparations of chitosan, crab chitosan, chitin (10 µg) were vigorously mixed with 50 µg of the different proteins and allow standing for 16 h at 32°C: S-layers from either 2362 or C7 strains, Bovine serum albumin (BSA). Free protein was determined and % bound calculated. Six independent experiments with duplicate samples were performed. Bars show the mean ± SD. Mann Whitney-U test was used to determine statistically significant differences between S-layers proteins and BSA control protein. *, P<0.05. With colloidal chitin, N-acetylglucosamine (NAG) (25 mM) was also added to verify binding inhibition. Five independent experiments with duplicate samples were performed. Mann Whitney-U test was used to determine statistically significant differences with and without NAG. **, P<0.05.

An *in vitro* assay was performed in order to check whether the pathogenicity of these S-layers involved the recognition of the chitin present in the cuticulum of their target. In a first assay, SDS-PAGE analysis of S-layers was performed in the presence or absence of colloidal chitin. Mobility retardation was observed for the S-layer but not for the bovine serum albumin (BSA) protein used as control (data not shown). To quantify the chitin binding property, a more sensitive assay was performed mixing purified S-layer proteins with insoluble chitosan, crab chitosan or colloidal chitin as described in Materials and Methods. As shown in Figure 3, the binding of S-layer proteins by these compounds featured similar efficacy, while this was not the case for BSA. Besides, the presence of N-acetylglucosamine (NAG), the monomer of chitin, inhibited the binding thus ensuring the specificity of the association between S-layers and chitin. Although binding to chitin was observed, we could not detect chitinase activity in the S-layer for these substrates.

### Hemolytic activity of S-layers

**Figure 4:**
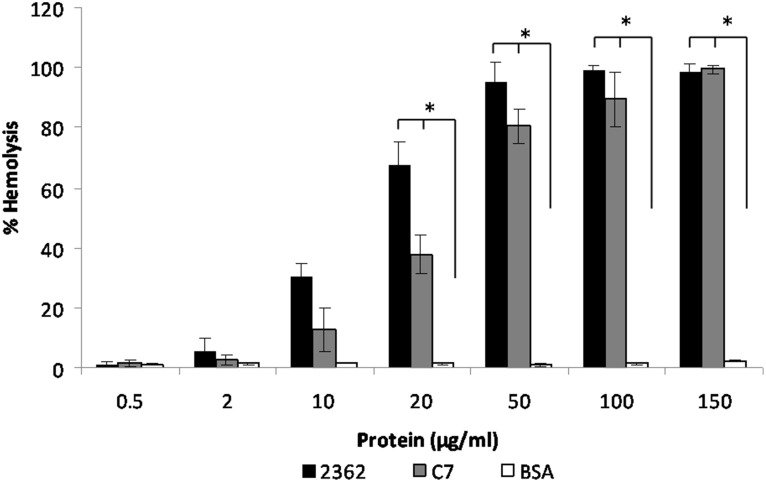
Hemolytic activity from S-layer of *L. sphaericus* 2362 and C7. Hemolysis was analyzed with 1% sheep red blood cells suspension in PBS. Bovine serum albumin (BSA) protein was used for unspecific effect. Six independent experiments with duplicate samples were performed. Bars show the mean ± SD. Mann Whitney-U test was used to determine statistically significant differences between S-layers proteins and BSA control protein. *, P<0.05.

We assayed hemolytic activity with purified S-layer preparation to determine the amount of hemoglobin release by the lyses of sheep red blood cells to obtain a direct quantitative measurement (see Materials and Methods). As shown in Figure 4, unlike BSA, the protein used as control, both S-layer proteins had hemolytic activity. We verified the specificity of the hemolysis by neutralizing the S-layer with specific antibodies that decreased the hemolytic activity to a 50%. We found that hemolytic activity was inhibited when N-acetylglucosamine was added, pointing to sugar recognition (data not shown).

## Discussion

The pathogenic role of the S-layer has already been shown for several *Bacillus* species like *B. cereus* and *B. anthracis* [23] [24], *Paenibacillus alvei* [25], different strains from *B. thuringiensis* [26] [27] [28] and *Lysinibacillus sphaericus* [29]. However, no function has been assigned to it other than its role as an adhesion factor for *B. anthracis* [23].

In the present work we confirm our previous observation [22] that spores from *L. sphaericus* retain S-layer proteins. Western Blot analyses of spore preparations from 2362 and C7 strains revealed associations of S-layer and Bin proteins (Fig. 1B). Immunofluorescence was used to visualize the S-layer (Fig. 2) surrounding spores. Since Bin proteins are deposited on the exosporium, we suspect that S-layer proteins are also associated to this structure. In fact, in *B. anthracis* the analysis of the exosporium shows the presence of several envelope proteins, including S-layers [35] [36] [42]. On the other hand, unlike the Mtx toxins present during the exponential growth, the S-layers were not degraded [16] by the proteolytic activity of the sporulation process, suggesting that their presence in spores was not fortuitous and that their location on the exosporium must contribute to this.

Moreover, the S-layer protein present in vegetative cultures (in the absence of spores), was mosquitocidal by itself as was the S-layer protein from new isolates reported by Lozano *et al* [29]. Concerning the larvicidal activity, it can be remarked that the S-layer from C7 was more active than that from 2362 against mosquitoes, in particular against *Aedes aegypti*, a species poorly sensitive to *L*. *sphaericus* toxins (LC50 was 10–times lower as shown in Table 1). We also reported a synergistic effect between cleaned spores (devoid of S-layer by EDTA treatment) and S-layers. When both are present, their activity is higher than that of the individual preparations (Tables 2).

The *slpC* gene from 2362 is 100% homologous to several entomopathogenic strains from the same antigenic group and especially to C3-41, a completely sequenced strain [43]. This led us to investigate functionalities within the protein sequence that could account for pathogenicity. Sequence based analysis reported in Material S1 revealed two possible biochemical domains: a chitin binding domain and a hemolytic domain that may contribute to the entomopathogenicity.

The chitin binding capacity (Fig. 3) and hemolytic activity (Fig. 4) were found to be present in the S-layer protein preparations and are both indicative of pathogenicity. Proteins with chitin-binding domains and chitinase activity have been described as pathogenicity factors in other species [44] [45]. In fact, the introduction of a chitinase gene from *B. thuringiensis* in a *L*. *sphaericus* strain increases its insecticidal activity [46]. The presence of a ubiquitous chitin-binding domain in an external protein would be a welcome feature that would facilitate its binding to chitin containing substrates such as insects, thus expanding the number of susceptible mosquitoes species. A chitin binding domain in the S-layer protein would favor the interaction and attachment to insects as an anchor for the bacterium to its potential host. It is worthwhile to remark that chitinases and chitin-binding proteins have been implicated as virulence factors in several species [45]. Since chitinases are enzymes with a large spectrum of substrates and not easy to characterize, we have not been able to detect any chitinase activity in *L*. *sphaericus* cultures nor in their S-layer using the substrates reported in Fig. 3 (insoluble chitosan, crab chitosan or colloidal chitin). Although the S-layer protein sequence analysis showed a putative chitinase active site within the Glyco_18 domain, the carboxi-terminal cleft responsible for the catalytic activity seems to be absent (Fig. S1). However, it is worthwhile stressing that *L. sphaericus*, a species known for its failure to transport and use glucose (hexoses) as carbon source, has an active PTS transport system and functions for the use of N-acetylglucosamine (NAG), the monomer of chitin [9] [10]. Since *L. sphaericus* share the same habitat as fungi able to degrade chitin and deliver NAG, we may speculate that an efficient PTS-NAG would ensure the survival of these bacteria in the same environment while the presence of the S-layer protein with its chitin binding capacity would favor their interaction and also the attachment to insects, a step necessary for the development of pathogenicity. In fact, the presence of NAG interferes with chitin binding as shown in Figure 3.

Furthermore, the S-layer protein was shown to have hemolytic activity. Also as for chitin binding NAG was found to interfere with hemolytic activity (not shown). Hemolysis is attributed to membrane distortions caused by proteins interacting with lipids. This capacity should help in the pathogenic effect within the larvae, probably contributing to bring down the number of resistant mosquitoes, in the same way the Cyt proteins act in *B. thuringiensis* strains.

*B. thuringiensis* Cyt and Cry toxins showed a synergistic pattern against *Aedes* larvae [47] [31] similar to our observations with S-layers and spores containing Bińs (Table 2).

The presence of S-layer in spore preparations has been reported for different *B. thuringiensis* strains [26] [27] [28] but their contribution to pathogenicity has not been confirmed. In such reports, the S-layer proteins were shown to be present as an inclusion inside the spore-crystals cytoplasm and were highly unstable, thus very difficult to isolate and characterize. Since we suspect that in *L. sphaericus* the S-layer is stabilized probably due to its location in the exosporium, as is the case in *B. anthracis*, it would be worthwhile to analyze it by cryo-EM [42].

Several strains belonging to the same antigenic group as 2362 presented S-layer proteins which cross-react with the antibody used in this work [30]; we also observed this cross-reactivity with that of the C7 strain, which implies they share a great homology. The reported sequences of the *slpC* gene from 2362 and C3-41 were identical. Moreover, the analysis of peptides obtained by trypsin digestion and MALDI-TOF analysis showed that the peptideś sequences of S-layer from 2362 and C7 were identical [48]. We wonder why the larvicidal activity was so variable among these strains presenting similar SlpC proteins. We cannot account differences between strains to chitin binding or hemolysis activities since the *in vitro* assay using mimetic substrates (chitin compounds and sheep red blood cells) might not be exactly identical to their target in mosquitoes larvae gut.

The absence of larvicidal activity of the *slpC* gene from C3–41 cloned into *E. coli* [49], would suggest that post-translational modifications of the S-layer taking place in the original host, might be necessary to ensure pathogenicity. A relationship between pathogenicity and S-layer post-translational modifications has been reported for other species [50] [51] [52].

In conclusion, we observed that the S-layer of *L*. *sphaericus* play several functions. In fact their presence during an osmotic stress is essential [48]. Besides, the S-layers, either from vegetative cultures or associated to spores, were larvicidal against mosquitoes; however that of C7 strain was more active and presented a wider spectrum of activity. The S-layer proteins high molecular weight could indicate that they might result from an assembly of several functional domains in a new protein. Also, multiple *slp* genes have been annotated for the C3-41 strain, suggesting the importance of this protein for this species.

Concerning the bioinsecticide activity, this is the first report of the presence of both toxins (BinA-B) and S-layer protein in spores of *Lysinibacillus sphaericus* strains, where both contribute to its pathogenicity. Together with the high capacity for metal biosorption of these spores [22], which must explain their higher survival in contaminated ponds [20], strains with a wider bioinsecticide spectrum such as C7, would be an interesting alternative to *B. thuringiensis* formulations.

The analysis of the post-translational modifications of the S-layer proteins, which may contribute to the proteins’ mosquitocidal activity and specificity, would be the aim of our future investigation.

## Supporting Information

Figure S1 **Sequence-based analysis. S1A:** Comparison of the structural disposition obtained with SMART SEARCH (Simple Modular Architecture Research Tool) for gb|AAA50256.1| surface layer protein [*Lysinibacillus sphaericus*] and dbj|BAM67143.1| chitinase [*Paenibacillus* sp. FPU-7]. **S1B and S1C:** EMBOSS Matcher Pairwise Sequence Alignment between gb|AAA50256.1| surface layer protein [*Lysinibacillus sphaericus*] and dbj|BAM67143.1| chitinase [*Paenibacillus* sp. FPU-7]. B) for the predicted Pfam SLH domains, C) for both predicted Internal Repeat with Glyco_18_1 domains. Symbols: “*” identical aminoacid, “:” indicates group similarity, “.” indicates low group similarity. Amino Acid Notation according to IUPAC-IUB-CBN. N° is for number of residues and position within proteins. Grey boxes are SLH domains. **S1D:** CLUSTAL O (1.2.0) sequence alignment between AAA50256.1 surface layer protein [*Lysinibacillus sphaericus* 2362, 1176 aa] and ACA38715.1 hemolysin-type calcium-binding domain-containing protein, *Lysinibacillus sphaericus*, C3-41, 874 aa): score 494.1 bits. Symbols: “*” identical aminoacid, “:” indicates group similarity, “.” indicates low group similarity. Amino Acid Notation according to IUPAC-IUB-CBN. N° is for number of residues and position within proteins. Grey boxes are SLH domains in AAA50256.1 only.
(DOCX)

Material S1 **Sequence-based analysis.** Free access sites were used to predict protein structure and function for the Surface layer protein AAA50256 [*Lysinibacillus sphaericus* 2362] and compared to possible orthologous proteins. URL links: **SMART**, (http://smart.embl-heidelberg.de/) Simple modular architecture research tool for Comparison of the structural disposition obtained with SMART SEARCH (Simple Modular Architecture Research Tool). **EMBOSS** Matcher Pairwise Sequence Alignment (http://www.ebi.ac.uk/Tools/services/web/toolform.ebi?tool=emboss_matcher&context=protein) identifies local similarities in two input sequences using a rigorous algorithm based on Bill Pearson’s lalign application. It enables to modify the default substitution scoring matrices (BLOSUM) for sequence alignment between distantly related proteins. **Clustal-O**, Global alignment tool (http://www.ebi.ac.uk/Tools/msa/clustalo/), Clustal Omega is a multiple sequence alignment program for proteins. It produces biologically meaningful multiple sequence alignments of divergent sequences.
(DOCX)
