# Supplementary material for: Contribution of S-Layer Proteins to the Mosquitocidal Activity of Lysinibacillus sphaericus
Source: PLoS One. 2014 Oct 29;9(10):e111114. doi: 10.1371/journal.pone.0111114 (PMC4213006; doi:10.1371/journal.pone.0111114)
Supplement: Figure S1 — Sequence-based analysis. S1A: Comparison of the structural disposition obtained with SMART SEARCH (Simple Modular Architecture Research Tool) for gb|AAA50256.1| surface layer protein [Lysinibacillus sphaericus] and dbj|BAM67143.1| chitinase [Paenibacillus sp. FPU-7]. S1B and S1C: EMBOSS Matcher Pairwise Sequence Alignment between gb|AAA50256.1| surface layer protein [Lysinibacillus sphaericus] and dbj|BAM67143.1| chitinase [Paenibacillus sp. FPU-7]. B) for the predicted Pfam SLH domains, C) for both predicted Internal Repeat with Glyco_18_1 domains. Symbols: “*” identical aminoacid, “:” indicates group similarity, “.” indicates low group similarity. Amino Acid Notation according to IUPAC-IUB-CBN. N° is for number of residues and position within proteins. Grey boxes are SLH domains. S1D: CLUSTAL O (1.2.0) sequence alignment between AAA50256.1 surface layer protein [Lysinibacillus sphaericus 2362, 1176 aa] and ACA38715.1 hemolysin-type calcium-binding domain-containing protein, Lysinibacillus sphaericus, C3-41, 874 aa): score 494.1 bits. Symbols: “*” identical aminoacid, “:” indicates group similarity, “.” indicates low group similarity. Amino Acid Notation according to IUPAC-IUB-CBN. N° is for number of residues and position within proteins. Grey boxes are SLH domains in AAA50256.1 only. (DOCX) [file pone.0111114.s001.docx]

**Figure S1: Sequence-based analysis**

1. SMART SEARCH

#=======================================

gi|556012|gb|AAA50256.1| surface layer protein [*Lysinibacillus sphaericus*]


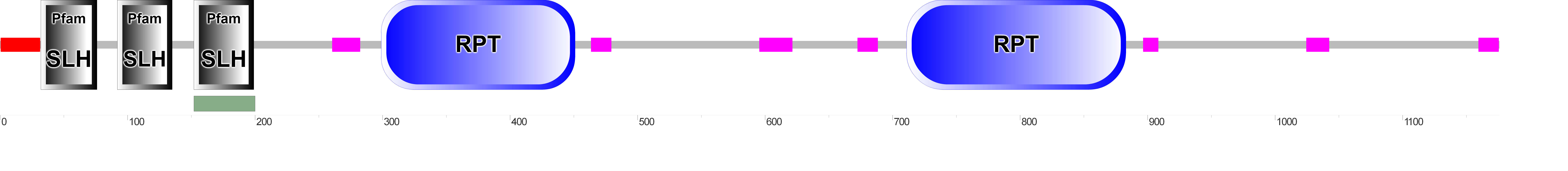


Pfam:SLH Position:32 to 76 E-value: 5.2e^-14^

Pfam:SLH Position:92 to 135 E-value: 3.4e^-15^

Pfam:SLH Position:152 to 199 E-value: 2e^-14^

Internal Repeat (prospero)IR_1 Position: 299 to 451 E-value: 8.787405e^-05^

Internal Repeat (prospero)IR_2 Position: 711 to 883 E-value: 8.787405e^-05^

#=======================================

gi|425029681|dbj|BAM67143.1| chitinase [*Paenibacillus* sp. FPU-7]


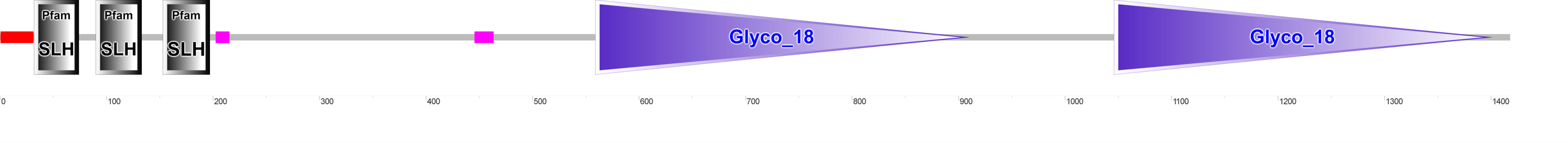


signal peptide Position: 1 to 30

Pfam:SLH Position:32 to 74 E-value: 2.1e^-8^

Pfam:SLH Position:90 to 133 E-value: 3.2e^-11^

Pfam:SLH Position:153 to 197 E-value: 5.6e^-11^

Glyco_18_1 Position:559 to 910 E-value: 8.57e^-108^

Glyco_18_2 Position:1046 to 1401 E-value: 1.19e^-101^

SLH Description: PFam PF00395 S-layer homology domain

IR_# Description: Internal Repeat detected by the Prospero program that compares protein sequences and profiles using the Smith-Waterman algorithm

Glyco_18_# Description: Interpro IPR011583 GH18(glycosyl hydrolases, family 18 or Glico_18)

SMART SM000636

#=======================================

| B) EMBOSS Matcher Pairwise Sequence Alignment:  # 1: BAM67143.1  # 2: AAA50256.1  # Matrix: EBLOSUM45  # Gap_penalty: 10  # Extend_penalty: 1  #=======================================  # Aligned_sequences: predicted Pfam SLH domains  # Identity: 55/201 (27.4%)  # Similarity: 100/201 (49.8%)  # Gaps: 11/201 ( 5.5%)  # Score: 276  #=======================================  AAA50256.1 8 RKFFAASATAALVASAIVPVASAAQLNDFNKISG-YAKEAVQSLVDAGVI 56  :*::......***.*:. .:****..:* :.* :*...:......*.*  BAM67143.1 9 KKWLVWCLCFALVFSSF-GMASAASTSD---VEGHWAAGTLNEWKSKGFI 54  AAA50256.1 57 QGDANGNFNPLKTISRAEAATIFTNALELEAEGDVNFKDVKADAWYYDAI 106  **.*:*:..*.:.::***...:...::.*....:*::.**.*..*.*..*  BAM67143.1 55 QGYADGSVKPDQAVTRAEFIALVNRSFALTETSEVHYSDVAASDWSYAEI 104  AAA50256.1 107 AATVENGIFEGVSATEFAPNKQLTRSEAAKILVDAFELEGEGD---LSEF 153  ....:.*..:*.......*:*:::*.**:.::....:**.... *:.*  BAM67143.1 105 GKAAKAGYIQGYEDGTIRPSKEISREEASVLVSRLIKLEHPSSHEILNTF 154  AAA50256.1 154 ADASTVKPWAKSYLEIAVANGVIKGSEANGKTNLNPNAPITRQDFAVVFSR 204  .*.:::..*:::.:......*:*:*.: **. :.*.*.***.:..*::.*  BAM67143.1 155 TDKASIASWSQAAVGALAEKGLIQGYD-NGV--FRPAASITRAESVVILDR 202  #======================================= | |
| --- | --- |
| C) EMBOSS Matcher Pairwise Sequence Alignment:  # 1: BAM67143.1  # 2: AAA50256.1  # Matrix: EBLOSUM45  # Gap_penalty: 10  # Extend_penalty: 1  # Aligned_sequences: predicted 153 aa Internal Repeat IR_1 with 352 aa Glyco_18_1  # Identity: 36/153 (23.5%)  # Similarity: 58/153 (37.9%)  # Gaps: 35/153 (22.9%)  # Score: 69  #=======================================  AAA50256.1 IR_1 7 YALKSGKTIKSVSLAADNK-TATVTLTDKLNNNKADAISISNVKAGDKEI 55  **:......*.*::..*.* *...*:*: **.*...* ...*.  BAM67143.1 GH18_1 30 YAFGRINNGKVVTIKEDAKWTEDPTITE------ADRIKRRN---NPDES 70  AAA50256.1 IR_1 56 NVKNVEFTAVDNKIPEVTEVKSLGTKAVKVTLSEPVENLSSTNFTLDGKA 105  *: ..:*.:..*.*.:..:.*:* ....*.:*...:*.:.:.  BAM67143.1 GH18_1 71 NL--AYLTGLKAKNPNLKVLVSIG--------GWEAEGFSDAALTPESRE 110  AAA50256.1 IR_1 106 YFGNVVMGAGNKTVILTPYSSSALSVGDHKLTVSGA--------KDFAGFVSL 150  .*.*..:...** *:...:.: *.:..*.** :*.*.*.:*  BAM67143.1 GH18_1 111 VFANSALDFMNK------YNLDGIDL-DWEYPVYGAWGVIKSRPEDKANFTAL 156  #=======================================  # 1: BAM67143.1  # 2: AAA50256.1  # Matrix: EBLOSUM45  # Gap_penalty: 10  # Extend_penalty: 1  # Aligned_sequences: predicted 166 aa Internal Repeat IR_2 with 356 aa Glyco_18_2  # Identity: 45/166 (27.1%)  # Similarity: 66/166 (39.8%)  # Gaps: 30/166 (18.1%)  # Score: 59  #=======================================  AAA50256.1 IR_2 12 DATVVT---ITFAETIKGDDVVFASGKAISGSGKVN-VNELQVMGVKDTS 57  ***.:* ..**. **.:.**..*.. :* ***:....:::..  BAM67143.1 GH18_2 21 DATKLTHINYAFAR-IKDNKVVKISED-------INWVNEFPSEEIREQR 62    AAA50256.1 IR_2 58 GNVHKKFNGSENKITLSSTSTPLK-LAKIDKDYDAKYTAE-LVDRKTVKV 105  .*.....*.:..* **...:..** *..* ..:.** :.*......  BAM67143.1 GH18_2 63 RNNPDDANFAYLK-TLKQQNPSLKVLVSI-----GGWAAEGFSDAALTPE 106  AAA50256.1 IR_2 106 KFSTVINSAAANAFTSESHKIDSIQVNGTSTV-----TVKFKDEINTNAS 150  ....:.***.* *..: :..*.*.::....* .:*.:.*...*.:  BAM67143.1 GH18_2 107 TREELANSAIA--FMHQ-YGFDGIDLDWEYPVYGAFGVIKSRPEDKQNFT 153  AAA50256.1 IR_2 151 DLDLKVNLSKLVDIAG 166  .* **:...** *:.*  BAM67143.1 GH18_2 154 AL-LKLFREKL-DVEG 167  #======================================= | |

D) CLUSTAL O (1.2.0) sequence alignment

ACA38715.1| ------------------------------------------------------------ 0

AAA50256.1 MAKQNKGRKFFAASATAALVASAIVPVASAAQLNDFNKISGYAKEAVQSLVDAGVIQGDA 60

ACA38715.1| ------------------------------------------------------------ 0

AAA50256.1| NGNFNPLKTISRAEAATIFTNALELEAEGDVNFKDVKADAWYYDAIAATVENGIFEGVSA 120

ACA38715.1| ------------------------------------------------------------ 0

AAA50256.1| TEFAPNKQLTRSEAAKILVDAFELEGEGDLSEFADASTVKPWAKSYLEIAVANGVIKGSE 180

ACA38715.1| ------------------------------------------------------------ 0

AAA50256.1| ANGKTNLNPNAPITRQDFAVVFSRTIENVDATPKVDKIEVVDAKTLNVTLSDGTKETVTL 240

ACA38715.1| ---------------------MG-----LFFYFIIGTQTATNLKQVVVEFDGKVDPSTAA 34

. : : : :*****:*******.** .**

AAA50256.1| EKALEPNKETEVTFKIKDVEYKAKVTYVVTTATAVKSVSATNLKEVVVEFDGTVDKETAE 300

ACA38715.1| EAGNYALTGANDPVVDTAVVSEDGSTVTLTVVDKLENQSEYKLAVNNIKAGDKVINAKDL 94

:*.****...: :.:. :: * .*.*:*:.***:*:. :::.*:***** **.*::

AAA50256.1| DAANYALKSGKT--IKSVSLAADNKTATVTLTDKLNNNKADAISISNVKAGDKEINVKNV 358

ACA38715.1| KFKPLDNTVPTVTKVDALGNKTVRVQFSEPVKAAQTSQFQIDGKVVVGSIQTNLNTVI-- 152

:*. :**.:* **:*.:**.*:*:* :****: .:::* :***. .*.: .

AAA50256.1| EFTAVDNKIPEVTEVKSLGTKAVKVTLSEPVENLSSTNFTLDGKAYFGNVVMGAGNKTVI 418

ACA38715.1| --IKLSSALTDGEHTLTAEGTEDYNSFKTVKADTKFNVVEDKTAPTVSVVSASFEKVVLK 210

****: *:*.**..*::*: .* :::: :*:***** ****: .:*::*.*.*.

AAA50256.1| LTPYSSSALSVGDHKLTVSGAKDFAGFVSLNSTHEFKVVEDKEAPTVTEATATLETVTLT 478

ACA38715.1| FSEPV--EQVFASNIYWMQGGSKKQASSVKQLADDKYELTFNNDNKLVY--TTDLFVTNV 266

*** : : * ***:** .* ***:**..:::**:**::.*: .:* : ..*::* ::

AAA50256.1| FSEDIDMDTVKASNVYWKSGDSKKEASEFERIADNKYKFVFKGSEKTLPTGKVDVYVEDI 538

ACA38715.1| KDYSGNVIDKDTKVQVTPVIDQTRPEVISSTFVKDSNNKQITIKFTKSLDTDTAKKAANY 326

**** * * ***** *** ******** . * : ::* *.:.*:*::* ::* *:.**

AAA50256.1| KDYSDNKIAKDTKVTVTPEIDQTRPEVRKVTAL---DEKTIKVTFSKTVDGESAIKTGNY 595

ACA38715.1| VIKDKDGKVQPISSTVNVSKDKEVTITLLGSLKDNTDYTLSVTGVADNTTLKNVMLPYTT 386

.:**** ** :.... ***.: .* * * . : *:::. * * *.*:*.** **

AAA50256.1| TVKDKDDKVVSVDKVTVDSKDSKSVIIDLYSKVSVGENTITIKNVKDATKLNNTMLDYTG 655

ACA38715.1| TLSVKDVTPPELTSVTRLGSK--QLYVAFNEAMATSGDGSIVDTDKYTVTGPDGKK---L 441

.:: .* *: * . :* :: : *:: * :: *:.* .:* *. * :

AAA50256.1| KFTRSDKEGPDYEHVINADAKAKKVVLKFDKKMDAA---SLADYSNYLVKINDTLQTLSE 712

ACA38715.1| TIASFNVTQDAKGVILNF-----------NNELPLSGPNF----KVKVQLVKDLAGNHLK 486

:*::.*::**. * :.* . :** :::* *** :** *

AAA50256.1| DVATLSVSNDATVVTITFAETIKGDDVVFASGKAISGSGKVNVNELQVMGVKDTSGNVHK 772

ACA38715.1| DLTKE---VAV-----------TDQTATIINSVEATAKNKVVVKFSNPIQSLVQGDFTIN 532

.:. . ::: *: :.* . ::.* ****. *:* . ** :

AAA50256.1| KFNGSENKITLSSTSTPLKLAKIDKDYDAKYTAELVDRKTVKVKFSTVINSAAANAFTSE 832

ACA38715.1| GSEIAHNEISTDGKTVTFTLKTDLTEDVKGVNLTVKPSPSTVDVL--------------G 578

. :* ::. .***..:* ::. :.. ::*.*: * . **: *

AAA50256.1| SHKIDSIQVN-GTSTVTVKFKDEINTNASDLDLKVNLS-KLVDIAGNESTNNTPIAIKAG 890

ACA38715.1| KNITGGVSPVVADKIAPTVDTEKITAVANRDDQIKITFNEAVQL-TAGGKPESDFIVKDG 637

*: .*:***. : *.**. : *.:**.* : : * .:** *.

AAA50256.1| INLLDSVAPVVVGE--PVVDK----------ETITFTFSENLTSVSIGEVLSTDFTVTRV 938

ACA38715.1| LKSSDNNVKVTKVELDAADATNKTLILTLEKSL-VA--ATVEVTNPRFVEDLAGNI---I 691

...* :* : ..* *.*: :::**... ** .* ..* ::: * *: *

AAA50256.1| SDNKDLAIK----DYSVAIANNNQVVITLSDNREVATAYKVTAKNAKLITDDNGDKKNAI 994

ACA38715.1| AKIEATSVELDSKGAATTLEAAKTALNASIATADTTVAVPVTEGKAVGNKVEGSKAALTT 751

*.: *:. . ..: :*:**** ** .*:.*. . . ***.*. *:: *** ** .

AAA50256.1| ADFTKTTATKVEASGTLSLDAAKTNLNNEITKAKD-AKATGTEGTAATNQIVGSKDALQV 1053

ACA38715.1| AITTAKAVD-TTTATLKQLTDAKAALDAAVTKYNNAVVVAITPALGNVTLAAV-NDTDSS 809

** .*: *. *:***:******: * **:* ** * * *: * *. .*. :

AAA50256.1| AIDVAELVKNDTAATLQQLTDAKTDLTAAITAYNAAKVEDISSLLVAPDLVLGTTDNGTI 1113

ACA38715.1| TTVALTTDETLEVISADLTKVAAVVDSGNKVKVTHVAAGTSVITVNVLNAEGKVVKTGTF 869

* .. * ***:* * . ::* .. . : ** * * : * *:**: **:****.

AAA50256.1| TGFVAGTGETLKVTSDSAANVEVTDP--TGLAVTAKAKGEANILVQVLK-GDKVIKTGTV 1170

ACA38715.1| TVTAS- 874

.**.*

AAA50256.1| KVTVSE 1176
