# Supplementary material for: Contribution of S-Layer Proteins to the Mosquitocidal Activity of Lysinibacillus sphaericus
Source: PLoS One. 2014 Oct 29;9(10):e111114. doi: 10.1371/journal.pone.0111114 (PMC4213006; doi:10.1371/journal.pone.0111114)
Supplement: Material S1 — Sequence-based analysis. Free access sites were used to predict protein structure and function for the Surface layer protein AAA50256 [Lysinibacillus sphaericus 2362] and compared to possible orthologous proteins. URL links: SMART, (http://smart.embl-heidelberg.de/) Simple modular architecture research tool for Comparison of the structural disposition obtained with SMART SEARCH (Simple Modular Architecture Research Tool). EMBOSS Matcher Pairwise Sequence Alignment (http://www.ebi.ac.uk/Tools/services/web/toolform.ebi?tool=emboss_matcher&context=protein) identifies local similarities in two input sequences using a rigorous algorithm based on Bill Pearson’s lalign application. It enables to modify the default substitution scoring matrices (BLOSUM) for sequence alignment between distantly related proteins. Clustal-O, Global alignment tool (http://www.ebi.ac.uk/Tools/msa/clustalo/), Clustal Omega is a multiple sequence alignment program for proteins. It produces biologically meaningful multiple sequence alignments of divergent sequences. (DOCX) [file pone.0111114.s002.docx]

**Supplemental material S1: Sequence-based analysis**

Free access sites were used to predict protein structure and function for the Surface layer protein AAA50256 [*Lysinibacillus sphaericus* 2362] and compared to possible orthologous proteins. URL links:

**SMART**, (<http://smart.embl-heidelberg.de/>) Simple modular architecture research tool for Comparison of the structural disposition obtained with SMART SEARCH (Simple Modular Architecture Research Tool).

**EMBOSS** Matcher Pairwise Sequence Alignment (<http://www.ebi.ac.uk/Tools/services/web/toolform.ebi?tool=emboss_matcher&context=protein>) identifies local similarities in two input sequences using a rigorous algorithm based on Bill Pearson's lalign application. It enables to modify the default substitution scoring matrices (BLOSUM) for sequence alignment between distantly related proteins.

**Clustal-O**, Global alignment tool (<http://www.ebi.ac.uk/Tools/msa/clustalo/>), Clustal Omega is a multiple sequence alignment program for proteins. It produces biologically meaningful multiple sequence alignments of divergent sequences.
